# Implementing electronic data capture (EDC) training for site staff

**DOI:** 10.1186/1745-6215-16-S2-P41

**Published:** 2015-11-16

**Authors:** Mark Webster-Smith, Claire Paulding, Stephanie Burnett, Lisa Jeffs, Dalia Ismail, Carolyn McNamara, Sharon Ereira, Rebecca Lewis, Emma Hall, Judith Bliss, Claire Snowdon

**Affiliations:** 1 The Institute of Cancer Research Clinical Trials & Statistics Unit (ICR-CTSU), London, UK

## Background

ICR-CTSU introduced EDC in 2012 necessitating the development of EDC specific training materials for participating sites.

## Challenges

Training of site staff on EDC systems is essential to ensure data quality and returns are maximised. Guidance notes are issued to support standardised completion of case report forms. However, due to the technical nature of EDC, guidance notes alone provide insufficient support for most site staff. Provision of effective, consistent and timely EDC training across a trial's lifespan is a continuing challenge, particularly for large trials or those with long follow-up where site staff turnover is an added consideration.

## Development of training materials

ICR-CTSU created a custom EDC system user guide, developed training slides and included instructional text within the EDC system. Since the implementation of EDC, ICR-CTSU have developed short training videos covering key topics including logging in, record navigation, data entry and query response. The videos illustrate the EDC screen view and are accompanied by scripted voice over guidance. Training materials are accessible online via the ICR-CTSU website.

## Conclusions

Whilst customised EDC system user guides and in-built instructions form the basis of site staff training, supplementary technical training is required. This can be provided to site staff individually through webinars, teleconferences and centre initiation visits, however the online training videos provide a more consistent and accessible resource. This solution should reduce demand on ICR-CTSU resources for ongoing site training.

